# Genotoxic Exposure during Juvenile Growth of Mammary Gland Depletes Stem Cell Activity and Inhibits Wnt Signaling

**DOI:** 10.1371/journal.pone.0049902

**Published:** 2012-11-21

**Authors:** Kristine S. Klos, Soyoung Kim, Caroline M. Alexander

**Affiliations:** McArdle Laboratory for Cancer Research, University of Wisconsin School of Medicine and Public Health, Madison, Wisconsin, United States of America; B.C. Cancer Agency, Canada

## Abstract

Various types of somatic stem cell have been tested for their response to genotoxic exposure, since these cells are likely to be important to regeneration, aging and cancer. In this study, we evaluated the response of mammary stem cells to genotoxic exposure during ductal growth in juveniles. Exposure to the polycyclic aromatic hydrocarbon (DMBA; 7,12 dimethylbenz[a]anthracene) had no gross effect on outgrowth and morphogenesis of the ductal tree, or upon lobuloalveolar growth during pregnancy. However, by fat pad assay, we found that mammary stem cell activity was reduced by 80% in glands from adults that were exposed to genotoxins as juveniles. The associated basal cell lineage was depleted. Both basal and luminal cells showed a robust response to genotoxic exposure (including γH2AX phosphorylation, pS^15^p53 and pT^68^Chk2), with durable hyperproliferation, but little cytotoxicity. Since the phenotype of these glands (low basal cell fraction, low stem cell activity) phenocopies mammary glands with loss of function for Wnt signaling, we measured Wnt signaling in genotoxin-exposed glands, and found a durable reduction in the activation of the canonical signaling Wnt receptors, Lrp5/6. Furthermore, when mammary epithelial cells were treated with Wnt3a, DMBA exposure reduced the basal cell population and Lrp activation was ablated. We conclude that during active ductal growth, Wnt-dependent mammary stem cells are sensitized to cell death by genotoxin exposure. Our conclusion may be important for other tissues, since all solid tumor stem cell activities have been shown to be Wnt-dependent to date.

## Introduction

It is important to understand the specific response of somatic stem cells to genotoxic exposure, especially in comparison to the cell majority in tissues. Stem cell function is uniquely associated with regeneration, aging and wound repair responses, and these cells may serve as precursor cells during tumor development [1]. Various somatic stem cells have been tested for their response to genotoxic damage, including hematopoetic stem cells, neural stem cells, the epidermal stem cells of the follicular bulge, and melanocytes. In the examples studied to date, stem cells undergo a range of responses to genotoxic exposure, from resistance, to senescence, death by apoptosis, or differentiation. These responses likely illustrate the compromises that are made for each specific tissue to maximize success of the animal. Thus, the preservation of essential stem cells in tissues with a high turnover rate may come at the price of genetic integrity, and the resistance to tumor development offered by the elimination of mutant stem cells may be offset by premature aging [2], [3], [4], [5], [6], [7], [8].

In this study, we evaluated the response of mammary stem cells to genotoxic exposure during juvenile development. The cell-autonomous stem cell activity characterized (so far) for mammary gland copurifies with one of the two principal epithelial lineages, the basal(/myoepithelial) cell population [9], [10]; thus after dissociation of mammary epithelial cells from the mammary ducts, a single basal cell can regenerate a whole mammary gland. Cells from the luminal population (responsible for milk secretion and the perception of the dominant estrogen growth signal) cannot reconstitute mammary gland, but this population does include progenitors that can generate limited outgrowths, and function as unipotent stem cells *in vivo* [11]. The overall frequency of ductal basal stem cells in mammary gland is at least 1/1600 (results from this study, these frequencies vary from strain to strain, and are tentative given that cell dissociation is likely to compromise functional activity). These cells cannot yet be recognized *in situ*, since there is no marker that can distinguish stem cells from the rest.

There are two phases of growth in the mammary gland, one that establishes the ductal tree during peri-puberty, and another during pregnancy that serves to fill the space between the ducts with lobuloalveolar units. Neither basal nor luminal cells are “terminally differentiated” since both divide at about the same rate during these processes [12]. For this study, we tested the effect of genotoxic exposure during juvenile growth. The cells born during ductal outgrowth are long-lived, compared to the majority that are born and die during pregnancy and estrus.

For this study, we selected a representative of the polycyclic aromatic hydrocarbons, DMBA (dimethylbenz[a]anthracene) as the genotoxin. This group of compounds are environmental genotoxins that form DNA adducts and induce double strand DNA breaks. DMBA is especially relevant to the study of mouse mammary gland, since it can be highly carcinogenic when administered later to adult female mice [13], [14], [15], [16]. Although DMBA-treated juvenile mammary glands showed a robust DNA damage response, gland outgrowth was normal. However, closer inspection revealed that the somatic basal epithelial stem cell activity was durably reduced by early exposure. The evident similarity between this phenotype and the phenotype of glands with loss of function of Wnt signaling [17], [18] led us to assay Wnt signaling activation in DMBA-treated glands. We found that Wnt receptor activation was reduced (for weeks, reflecting the loss of stem cell activity). Furthermore, we found that when basal mammary epithelial cells were actively accumulating in response to Wnt signaling *in vitro*, they were highly sensitized to cell death in response to genotoxic exposure. We propose that this mechanism accounts for the durable loss of stem cell function observed after exposure of young mice to genotoxins.

## Materials and Methods

### Mice

*BALB*/c mice were housed according to the regulations set by the University of Wisconsin Institutional Animal Use and Care Committee. DMBA was dissolved in tricaprylin (Sigma-Aldrich, St. Louis, MO)(5 mg/ml) and injected intraperitoneally into virgin juvenile (35-day-old) *BALB*/c females (0.10 µmol or 25 µg DMBA/g bdwt mouse). For measurement of their acute response to radiation exposure, mice were subjected to 10 Gy (Mark I ^137^CsCl irradiator, J.L. Shepherd & Associates, San Fernando, CA), and mammary glands collected 30 minutes later. To label mitotic cells, 5-bromo-2′-deoxyuridine (BrdU, Sigma-Aldrich) was injected (0.1 mg BrdU/g bdwt mouse) and glands collected two hours later.

### Whole Mount Staining and Immunohistochemistry

Mammary glands were fixed and processed to visualize ductal trees as described [17]. General methods for immunohistochemical analysis of paraffin-embedded mammary samples include antigen retrieval as described [17]. Primary antibodies are: anti-Ki67 (BD Biosciences, Franklin Lakes, NJ), Keratin 5 Clone AF 138 (Covance, Emeryville, CA), Keratin 8 (The University of Iowa – Developmental Studies Hybridoma Bank, Iowa City, Iowa), γH2AX (Millipore, Billerica, MN), BrdU (Roche, Indianapolis, IN), phospho-Ser^15^ p53 (Cell Signaling Technology, Beverly, MA). Secondary antibodies and reagents were: AlexaFluor 488 goat anti-mouse, AlexaFluor 546 goat anti-rat, AlexaFluor 488 goat anti-rabbit, AlexaFluor 647 goat anti-rabbit, Pacific Blue conjugated goat anti-rabbit (all from Invitrogen, Carlsbad, CA). To calculate the mitotic index of basal and luminal cells, paraffin sections of mammary glands from BrdU-injected mice were counterstained with lineage-specific markers (K5 and K8), together with a nuclear stain (to score the total cell number of each cell type). To describe the focalized nature of the excess mitotic index in genotoxin-exposed glands, we counted “bursts” as five or more BrdU positive cells in a 20-cell radius.

### Preparation of Mammary Epithelial Cells (MECs), Fat Pad Stem Cell Assay, Primary Culture Methods and Culture of HC11 Cells

Primary MECs were isolated as described and transferred to fat pads in 3 week-old recipient mice [17]. Data from limiting dilution assays were tested for statistical fit of clonogenic activity (using a likelihood ratio test), and stem cell frequencies were estimated from the LimDil statistical program (http://bioinf.wehi.edu.au/software/limdil). To transfer MECs to primary culture, Lab-Tek II 4-well chamber slides (Thermo Fisher Scientific Inc, Waltham, MA) were coated with growth factor-reduced Matrigel (precoated plates with 0.7 mg/ml; BD Biosciences) and cells were plated in DMEM/F12 (Invitrogen) containing 2% FBS (Harlan Laboratories, Indianapolis, IN), 10 µg/ml insulin (Sigma), and 100 U/ml Penicillin/Streptomycin (Invitrogen), with 20 ng/ml EGF and/or 100 ngs/ml rmWnt3a (R&D System, Minneapolis, MN). HC11 cells were grown in RPMI media (Invitrogen) supplemented with 5–10% FBS (Harlan), 5 µg/ml insulin (Sigma-Aldrich), and 10 ng/ml EGF. For immunocytochemical assay, cultured cells were rinsed with PBS, fixed with 2% PFA (Electron Microscopy Sciences) for 15 min, permeabilized with 0.1% or 0.5% Triton-X100 (depending on non-nuclear/nuclear antigen; 5 min) and blocked with 10% goat serum for 1 hour (Jackson ImmunoResearch Labs).

### Flow Cytometric Analysis

MECs (1×10^∧^6) were incubated with 1.0 µg/ml CD31-APC, 1.0 µg/ml CD45-APC, 0.6 µg/ml CD24-PE, and 30 µl/ml CD49f-FITC (all from BD Biosciences) in Hanks’ Balanced Salt Solution Modified (Stem Cell Technologies) plus 2% FBS (Harlan) for 1 hour on ice. Cells were fixed in 1% paraformaldehyde (Electron Microscopy Sciences, Hatfield, PA) for 30 min at 4°C, permeabilized with 0.1% Triton-X-100 for 15 min at RT, and then incubated with 4′,6-diamidino-2-phenylindole dihydrochloride (DAPI, 20 ug/ml; Invitrogen) 30 min before flow analysis. The cell suspension was analyzed on an LSRII (BD Biosciences) equipped with FlowJo software (Tree Star Inc. Ashland, OR). The gating profiles and trees are shown in Fig. S1A and B, and were generated according to [19].

### Cell and Tissue Preparation for Western Blot Analysis

Cells were rinsed with PBS and scraped into a high salt lysate buffer (25 mM HEPES pH 7.4, 300 mM NaCl, 1.5 mM MgCl_2_, 1 mM EGTA, and 0.5% Triton X100, with Halt protease and phosphatase inhibitor cocktails, Pierce, Rockford, IL). For tissues, mammary glands were snap frozen in liquid nitrogen and ground with a mortar and pestle. The resulting powder was mixed with high salt buffer containing protease and phosphatase inhibitors and homogenized for ten seconds, twice, at 4°C with a Polytron (Kinematica, AG, Switzerland). The resulting tissue and cell lysates were cleared for 20 min at 10 000 g at 4°C and the supernatant was analyzed by Western blotting. Antibodies used as probes were: anti-phospho-Lrp6 (detecting both phospho-Lrp6 and phospho-Lrp5; Cell Signaling Technology), anti-vinculin (Millipore), and anti-β-actin (clone AC-15, Sigma-Aldrich). Secondary antibodies were peroxidase-conjugated anti-mouse IgG (Jackson ImmunoResearch Laboratories, West Grove, PA) and horseradish peroxidase conjugated anti-rabbit IgG (InVitrogen).

## Results

Growth and development for the mammary gland is stimulated in two waves, the first occurs during pre-puberty (1–8 weeks of age, depending on the strain), when the ductal tree branches out from the placode to colonize the mammary fat pad. The second describes the full development of the lobulo-alveolar lineage during pregnancy. The vast majority of cells that develop during pregnancy involute at the end of lactation, whereas the majority of ductal epithelial cells are likely to be long-lived in mice and women. To examine the interaction of environmental genotoxins with mammary gland, we tested the effect of early exposure of these growing ductal populations to the polycyclic aromatic hydrocarbon, DMBA, measuring DNA damage repair and checkpoint activation 1 and 2 days after exposure, and subsequently the effect of this exposure on the differentiation and function of mammary glands (2 and 7 weeks afterward).

**Figure 1:**
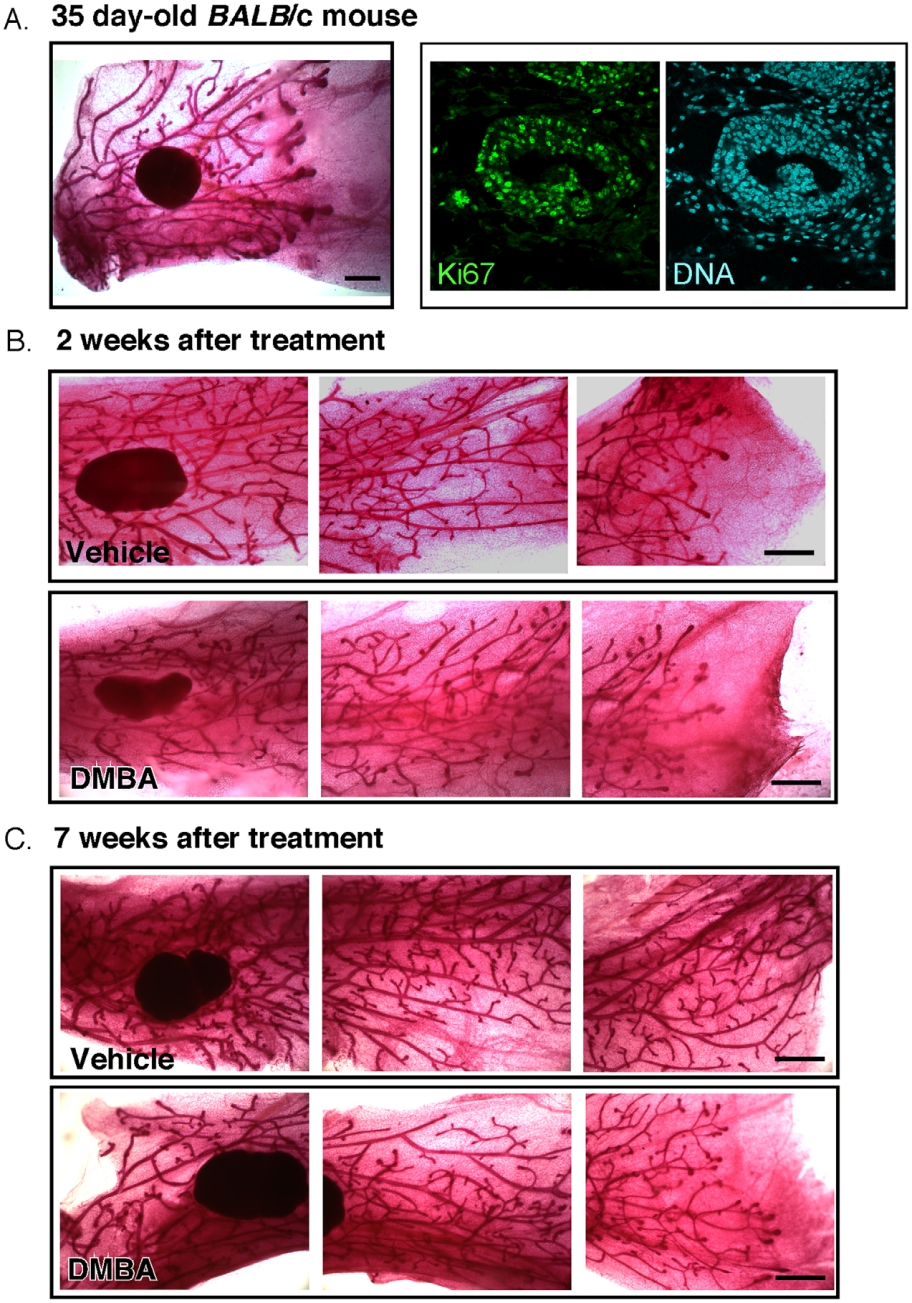
Carcinogen exposure during juvenile development does not significantly alter ductal tree growth or morphogenesis during puberty or pregnancy. (A) *Ductal outgrowth.* Whole mount preparation of a representative mammary ductal tree in 35 day virgin female *BALB*/c mice, growing from the nipple (left hand side) rightward past the lymph node (dark inclusion). On this day, mice were injected intraperitoneally with 0.10 µmol DMBA/g mouse or vehicle (tricaprylin). Scale bar = 1 mm. To evaluate the mitotic index of mammary epithelial cells at this stage of development, paraffin sections of glands were stained with anti-Ki67 (or a nuclear counterstain). The multi-layered epithelium shown is typical of terminal end bud. Scale bar = 50 µM. (B, C) Whole mount preparations of mammary ductal trees harvested from mice treated with vehicle or DMBA, 2 or 7 weeks after treatment (as indicated).

At 5 weeks of age, the mammary ductal trees of female BALB/c mice were actively extending (shown by dilated terminal end buds), and showed high rates of cell division (Fig. 1A). Female mice were administered DMBA (see Methods) at a dose shown previously to induce DNA adduct formation, to activate DNA repair and to induce mutational changes [20]. Gross development of mammary ductal trees was assessed 2 and 7 weeks later, by whole mount staining of mammary glands (to assess morphogenesis and rate of outgrowth). DMBA-treated glands were almost indistinguishable from untreated mice and mice administered vehicle (Fig. 1B,C). (In fact, administration of the vehicle, tricaprylin (approximately 100 µl of this synthetic triacylglyceride), induced a significant increase in ductal branching (48%), with or without DMBA; data not shown).

**Figure 2:**
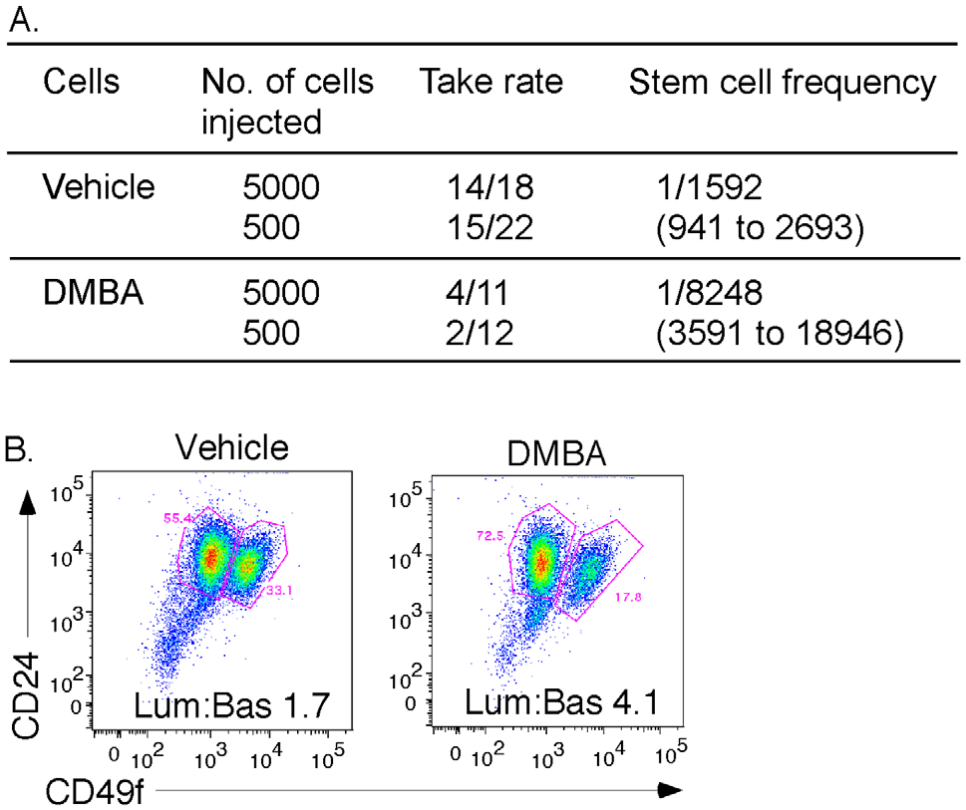
Genotoxin exposure during juvenile development affects differentiation and stem cell frequency in adult ductal trees. (A) *Stem cell assay.* Mammary epithelial cells populations were prepared from adults (9–10 weeks old), either exposed to DMBA at 5 weeks or not (administered tricaprylin vehicle), and injected into cleared fat pads at various limiting cell numbers. Four to six weeks later, glands were assessed for colonization and scored as a take (more than 25% colonization), or no take. The data fit the limiting dilution model (see Methods section; likelihood ratio test of single-hit model: *P*<0.000001) and stem cell frequencies were estimated on the basis of the LimDil statistical program (difference between groups: *P*<0.0001). (B) *Flow cytometric analysis of MEC populations from adults exposed to DMBA as juvenile mice.* Mammary epithelial cells were dissociated from 3 mice each (DMBA-treated and control), and stained according to [9], [17], to resolve basal and luminal epithelial cell populations (see Fig. S1 for gating details). Representative flow cytograms are shown. The two principal cell types, luminal and basal, were quantified, and the ratio of luminal/basal cell is shown.

There are published studies that suggest that somatic stem cells are unusually resistant to genotoxin administration (compared to other more differentiated cell types). We therefore tested the mammary stem cell activity of adult female mice that were administered DMBA as juveniles. Limiting dilutions of mammary epithelial cells were transferred into fat pads *in vivo* (Fig. 2A). The stem cell frequency in adult glands was significantly lower than normal, only 1/8300 compared to 1/1600, equivalent to a loss of 80% of stem cell activity. Previous work from our lab has shown that depleted basal epithelial stem cell populations are associated with depleted basal cell populations (compared to the total epithelial cell population [17]). We tested the relative differentiation of MECs using the flow cytometric protocol (established by Stingl et al [9]) that we previously characterized. This separates luminal and basal cell populations to measure their relative number. For DMBA-treated mammary glands, we found that the normal luminal: basal ratio (1.7) was increased to 4.1, which is a ratio more typical of stem cell-deficient glands (Fig. 2B).

**Figure 3:**
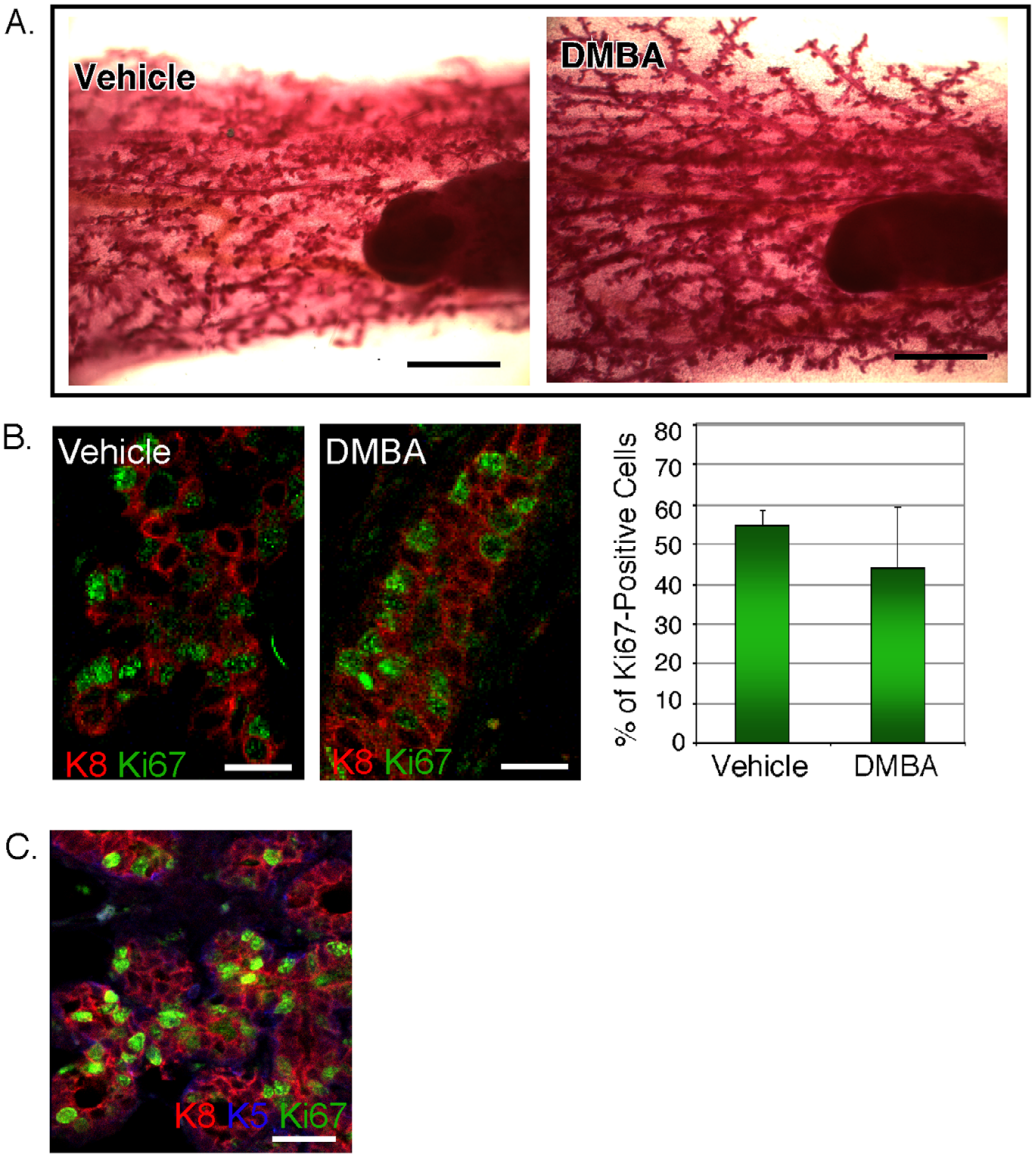
Development of genotoxin-exposed glands during pregnancy. (A, B) *Analysis of lobulo-alveolar development during pregnancy.* Whole mount preparations from timed pregnant mice (6d p.c, 10 week old mice), exposed as juveniles to DMBA (or not). To examine the pattern of growth in more detail, paraffin sections from mammary glands were immunostained with Ki67 (to show cells in cycle; green) and counterstained with a luminal cell-specific stain, CK8 (K8; red; scale bar = 25 µm). Quantitation of the Ki67 index showed no significant difference between genotoxin-exposed mice and the control cohort. (C) *Lobulo-alveolar development during pregnancy in Lrp5−/− glands.* Samples were processed according to (B), and were similarly stained with Ki67 and CK8, and also with CK5 (K5; blue) to visualize basal epithelial cells.

We evaluated mammary development during pregnancy in mice exposed to genotoxin as juveniles. Timed pregnant glands were removed from treated and untreated mice and assessed for their gross lobuloalveolar development, by whole mount staining (Fig. 3A) and mitotic index assay (Fig. 3B). This revealed that the growth and differentiation associated with pregnancy was unaffected in mammary glands that were exposed to genotoxins during early development. Other stem cell-deficient glands also show normal development associated with pregnancy, including those with mutations in Lrp5 or β1 integrin [17], [21]. The mitotic pattern of an *lrp5−/−* mammary gland from a pregnant mouse is shown in Fig. 3C.

**Figure 4:**
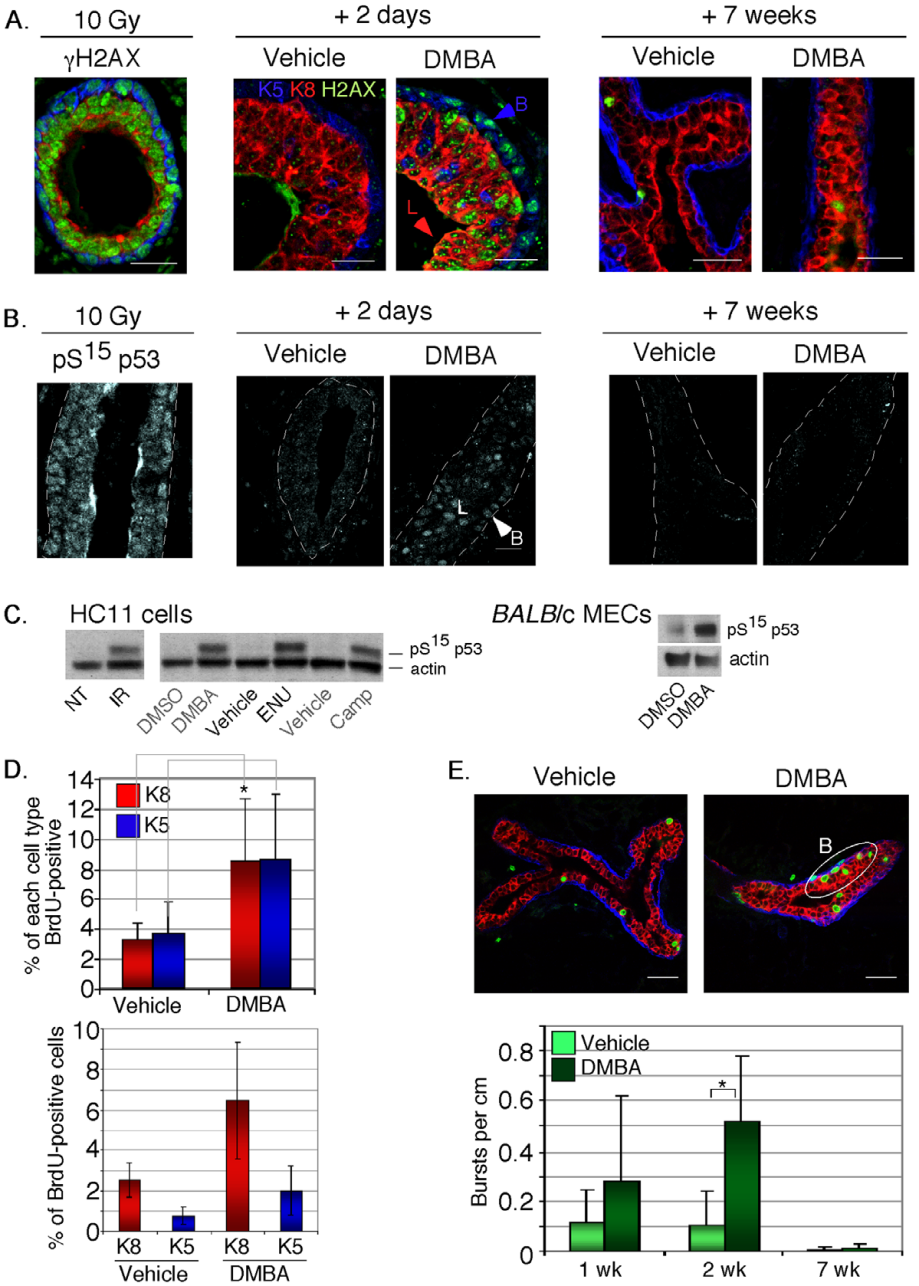
Evaluation of the DNA damage response (DDR) in basal and luminal epithelial cells after genotoxin administration *in vivo*. (A) *DNA damage focus assembly.* Mice were treated with either 10 Gy γ-irradiation and harvested 30 minutes later (positive control), or administered DMBA (2 µg/ml) or vehicle (tricaprylin), and their mammary glands were harvested 2 days or 7 weeks later (as shown). Paraffin sections were tested for the formation of nuclear-associated γH2AX foci (green) in basal and luminal epithelial cells (K5, blue and K8, red respectively, counterstained with TOPRO). (B) *DDR checkpoint activation.* Similar sections were evaluated for their activation of p53, by assaying the nuclear accumulation of pS^15^-p53. Basement membranes are demarcated by the dotted line, and examples of nuclei positive for pS^15^-p53 are shown (B, basal; L, luminal). (C) *pS^15^-p53 activation in response to DMBA.* To evaluate the activation of DDR in response to DMBA, compared to other more canonical genotoxins, lysates of mammary epithelial cells (HC11 cells) treated with various genotoxins, were evaluated by Western blotting. The genotoxins were: 10 Gy irradiation (1 hour), 2 µg/ml DMBA (24 hours; this is a pro-genotoxin requiring activation by metabolism, or vehicle, DMSO), *N*-ethyl-*N*-nitrosourea (1 hour after addition of 500 µg/ml ENU, a direct acting alkylating agent, or control PCB), or 20 µg/ml camptothecin (1 hour, camptothecin is a topoisomerase I inhibitor, or DMSO). Similarly, the specificity of the nuclear stain was cross-checked to Western blot data of lysates of primary mammary epithelial cells in culture, exposed to DMBA (as indicated). Non-treated cells (NT) are also shown for comparison. (D) *Induction of proliferation is equally allocated to basal and luminal cells after exposure to DMBA.* Genotoxin-exposed and control-treated mice (1, 2 and 7 weeks after treatment) were administered BrdU (intraperitoneally), and mammary glands harvested 2 hours later. Data that describes the lineage-specific mitotic index is shown for samples 2 weeks after DMBA treatment. Thus, the fraction of BrdU-positive basal and luminal cells was assayed (n = 3, >2000 cells each), and illustrated here as a fraction of each cell type (top panel) or as a fraction of total cells (basal cells are a minority). Statistically different values are indicated * (p<0.05). (E) *Induction of proliferation is focalized after exposure to DMBA.* Genotoxin-exposed and control-treated mice (1, 2 and 7 weeks after treatment) were administered BrdU (intraperitoneally), and mammary glands harvested 2 hours later. Mitotic cells were widely distributed in vehicle-treated glands. Though this pattern was also evident in DMBA-administered glands, in addition, there were “bursts” of mitotic activity, defined as five or more BrdU positive cells in a 20-cell radius (B). Bursts were measured per length of duct (n = 3; arbitrary unit of cm after image capture).

**Figure 5:**
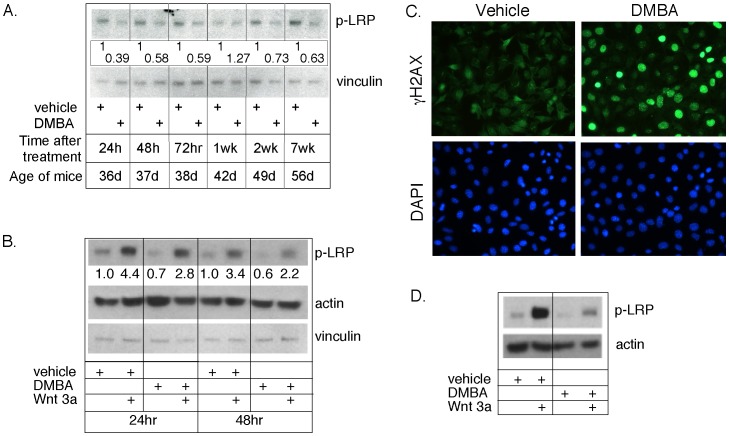
DMBA-induced DDR is accompanied by loss of Wnt signaling *in vitro* and *in vivo*. (A) *DMBA exposure reduces LRP activation in vivo, and this reduction in active Wnt signaling is durable*. Mammary glands from DMBA-treated (and control) mice were snap-frozen, ground and lysed as described. Proteins were analyzed by Western blotting, and assayed for activation of LRP (p-LRP). Blots were normalized using a vinculin internal loading control. The relative signal was calculated for each pair of treated/control samples, for the timepoints indicated post-exposure. (B) *DMBA exposure of MECs in vitro reduces Wnt ligand -induced (and basal) activation of Wnt signaling.* MECs were transferred to culture for 24 h, treated with Wnt3a (100 ng/ml, or not) as described in the Methods section, and lysed for analysis of p-LRP, 24 and 48 hours later. (C, D) *DMBA exposure of HC11 mammary epithelial cells reduces Wnt ligand -induced (and basal) activation of Wnt signaling*. (C) HC11 cells were treated with DMBA and assayed for the appearance of γH2AX 24 hours later (by immunostaining as detailed for Fig. S3). (D) Lysates of similarly treated cells were analyzed by Western blotting for LRP activation (phosphor-LRP).

**Figure 6:**
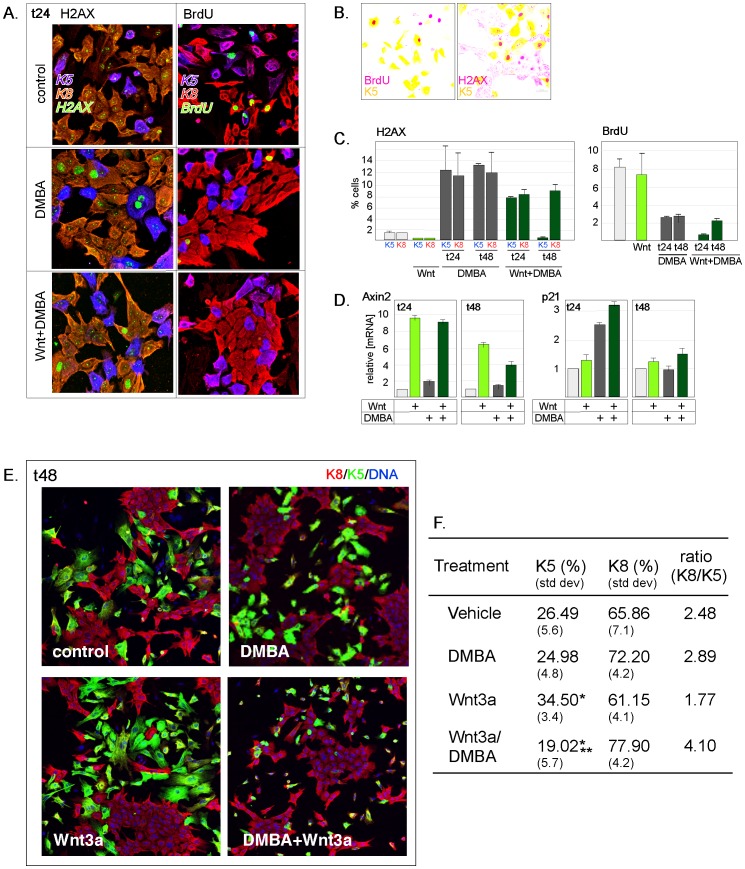
The genotoxic response ablates Wnt-induced basal cell accumulation. (A) *Immunohistochemical analysis of lineage-specific responses to DMBA and Wnt3a.* Cultured MECs were transferred to culture for 24 hours, and treated with DMBA (2 µg/ml), recombinant mouse Wnt3a (100 ngs/ml) or both for 24 hours, as indicated (Wnt alone, data not shown). Cells were either incubated with BrdU (for 60 mins) or not, then fixed and stained as indicated. (B) *Examples of images used for quantitative analysis*. To obtain the fraction of cells of each lineage that showed activation of DNA damage, or cell division, the image of the lineage-specific marker was overlaid on H2AX or BrdU. (C) *Quantitation of immunohistochemical analysis of lineage-specific responses to DMBA and Wnt3a.* The fraction of each of K5- or K8-positive cells was scored for expression of H2AX, or mitotic index, 24 or 48 hours after exposure to DMBA or Wnt3a (as indicated). (D) *Assay of Wnt- and DDR-dependent transcriptional reporters*. The Wnt reporter, Axin2, and the p53 target, p21, were measured by qPCR assay of RNA extracts of cell lysates. (E). *Visualization of luminal and basal cells in Wnt-treated cultures.* Cell cultures were fixed and stained with lineage-specific markers (K8, luminal; K5, basal) together with a nuclear counterstain. (F) *Quantitation of lineage-specific responses to DMBA and Wnt3a*. Results were quantified (at least 1500 cells were counted in each sample; 3 experiments), and the relative differentiation of cultures expressed as % K5 and K8-positive cells, and the luminal/basal ratio. * indicates significant difference from non-Wnt3a treated cultures, and ** indicates significant difference from both non-Wnt3a treated, and Wnt-treated cultures.

We considered the possibility that the relative loss of basal cells (together with the basal stem cell activity) may reflect a lineage-dependent susceptibility to cell death after exposure to genotoxin administration. Lineage-specific sensitivities to DNA damage have been observed before [22], [23], [24]. First, we aimed to test whether the genotoxin was perceived equally by both lineages *in vivo* (DMBA is required to be metabolized to the proximal mutagen). Mice were irradiated with 10 Gy, to test whether the DNA damage repair (DDR) response was present in all cells. After treatment, we found that all mammary epithelial cells were competent to respond (within the limits of detection; Fig. 4A). The formation of γH2AX nuclear foci can be used as a quantitative measure of the cellular response to double strand DNA breaks, which form as a repair intermediate [25], [26]. Using mammary epithelial cell lines, we confirmed that γH2AX focus formation was an effective assay for DDR activation after exposure to non-cytotoxic doses of DMBA (Fig. S2). (In fact, DMBA was as effective at inducing γH2AX foci as the other 3 double strand-break inducing agents used for comparison; Fig. S2A). After DMBA exposure *in vivo*, γH2AX foci formed in a sub-population of basal and luminal cells (Fig. 4A), and these were gone 7 weeks later. Interestingly, not all mammary epithelial cells showed activated γH2AX after DMBA exposure, indeed only 10–20% MECs stained positive, both *in vivo* (Fig. 4A) and *in vitro* (Fig. 5C, HC11 cells; Fig. 6C, MECs).

We also tested the hypothesis that DDR checkpoints are selectively activated in basal or luminal cells. Firstly, since the p53 pathway has been shown to be a key mediator of the DNA repair response in mammary gland and breast tumors [27], [28], [29], we measured p53 activation, using nuclear localization of phospho-S^15^p53 (Fig. 4B). The specificity of this induction was also illustrated *in vitro* by Western blot (Fig. 4C), whereas another activated p53 species, phospho-S^20^p53, was constitutively nuclear *in vitro* (Fig. S2B). (Brca1 staining was ubiquitous in all cells, showed nuclear localization, and was present irrespective of genotoxic challenge; Fig. S2C). Both γH2AX and p53 showed significant rates of spontaneous activation in culture media without EGF supplementation (Fig. S3A), and this stress was higher for basal cells (18% versus 8% in luminal cells), with and without DMBA exposure. However, this culture-associated genotoxic stress was almost eliminated by the addition of EGF to the cultures (Fig. 6C), and with EGF, both luminal and basal cells showed equal numbers of γH2AX-positive cells in response to DMBA.

We evaluated the relative response of basal and luminal cells to DMBA, 24 and 48 hours after treatment, counting the cell number of each type. In the absence of EGF, luminal cells were more sensitive to DMBA-induced cell death in these culture conditions, scored either from cultures of the total population, or after purification of luminal cells followed by mono-culture (Fig. S3D, E). (This latter experiment was done to evaluate whether activation of DDR changed the lineage specification of luminal cells). In the presence of EGF, both cell types showed similar responses (approximately 30% of both basal and luminal cells showed p53 activation after DMBA exposure). Overall, these results suggested that culture conditions can modulate the outcome of genotoxicity assays in a lineage-specific manner.

To cross check this result in vitro to the prevailing regulation *in vivo*, we evaluated mammary glands exposed to DMBA. We found that there were broadly similar fractions of basal and luminal cells (up to 30%) with nuclear pS^15^-p53 (Figs 4A,B), and determined that this checkpoint was fully resolved within 7 weeks of treatment. We also tested MECs *in vitro* for their relative activation of the Atm-Chk2-p53 pathway (Fig. S3). Chk2 is a cell cycle checkpoint protein likely to be functionally important to damage repair, and mutations in Chk2 are associated with a higher risk of breast cancer in humans. Approximately 10% of cells, either basal or luminal showed activated Chk2, assayed as nuclear p-T^68^Chk2. Overall, the data describing these DDR response indicators did not support a significant difference in primary response proteins in the two primary lineages.

Interestingly, we did observe a prolonged and significant proliferative response to DMBA administration. Thus, both lineages showed a 2–3 fold increase in their mitotic index 1–2 weeks after treatment (Fig. 4D, E), which normalized 7 weeks afterward. This proliferative response was focalized into cell groups (we termed “bursts”, defined as mitotic cells clustered within a 3 cell distance of other mitotic cells). These cell groups comprised both basal and luminal cells. This suggests that there are groups of cells that show persistent hyperplasia after the DDR response is complete. Perhaps paradoxically, DMBA administration at these doses did not induce rates of cell death that are measurable *in vivo* (2 days, or 1, 2 or 7 weeks; data not shown). This was also observed for irradiated explants of human breast [22], and may explain why rates of outgrowth are normal for these ductal trees (Fig. 1).

Since our data suggested that DDR activation was approximately equivalent for basal and luminal cells, but responses could be modified by the signaling pathways activated in each cell lineage, we turned to a signaling pathway known to be important to the physiology of stem cells. Thus, we have previously shown that basal mammary stem cell activity depends upon Wnt signaling for induction (or maintenance) *in vivo* [17]. We therefore tested the interaction of Wnt signaling with the response to genotoxic exposure *in vivo* and *in vitro.* When lysates of mammary glands were analyzed for the presence of activated canonical Wnt receptors (using an anti-phospho Lrp antibody), activation was reduced by at least half during the acute response phase, and this reduction persisted even after the DDR response was resolved (7 weeks later; Fig. 5A). When MECs were transferred to culture, and assessed for their relative activation of Lrp in the presence and absence of DMBA, both the basal and induced activation (induced by the addition of 100 ng/ml Wnt3a) was reduced (by approximately 2×; Fig. 5B). For HC11 cells, a mammary epithelial cell line with a mutant p53 species, Wnt signaling responses were also inhibited by 80% in the presence of DMBA (Fig. 5 C,D).

Only basal epithelial cells express Lrp proteins, and are predicted to respond to Wnt ligands with a canonical Wnt signal *in vivo.* In order to test whether Wnt signaling could explain lineage specific responses to genotoxic exposure, MECs were cultured with or without Wnt3a and DMBA, and the activation of the DDR was measured in basal and luminal cells (triple stains at 24 hours after DMBA exposure; Fig. 6A), together with the effect of this on the mitotic index of each lineage. Fig. 6B illustrates the overlaid dual-stained images analyzed to generate the quantitative information shown in Fig. 6C. Approximately 12% of luminal or basal cells showed activation of the DDR, 24 and 48 hours after DMBA exposure. In the presence of Wnt ligand, the overall rate of growth was not significantly different (Fig. 6C). However, although Wnt did not change the relative ability of luminal and basal cells to activate H2AX throughout the activation phase, these two cell types showed vastly different responses during the cellular response phase (48 hours after treatment). Indeed, there were no remaining basal cells with nuclear H2AX (Fig. 6C). This suggests that only Wnt-responsive basal cells (and not basal cells in the presence of EGF/2% serum) showed a selective cell death response to the DDR. The activation of canonical Wnt signaling was assessed by assay of Axin2 mRNA (Fig. 6D), and this was not suppressed by DDR/DMBA administration. As a marker of p53 activation, p21 mRNA expression was analyzed, and showed significant induction in cultured cells 24 hours after exposure to DMBA. During the response phase, cells expressing p21 were eliminated.

Basal cells were observed to accumulate in the presence of Wnt3a, using lineage-specific stains (Fig. 6E). Typically, basal cells comprised approximately one out of four cultured epithelial cells, and the ratio of luminal/basal cells was 2.5. The addition of DMBA had a non-statistically significant effect on the survival of basal cells in the absence of Wnt3a. Treated with Wnt3a, the relative frequency of basal cells increased (K8/K5 = 1.77). At least half the basal cells were gone when these cultures are treated with both DMBA and Wnt3a (K8/K5 = 4.10; Fig. 6F). Therefore, we conclude that under conditions where Wnt signaling is the prime factor in the survival, differentiation and growth of basal mammary epithelial cells, these cells cannot survive genotoxic exposure.

## Discussion

The effect of environmental agents on regenerative activity and stem cell function is important to understand. In this study, we have found that exposure of peri-pubertal female mice to the genotoxin, DMBA, does not affect gross function of mammary gland in these mice as adults (by any functional or morphological criteria). However, mammary stem cell activity is depleted by 80%. This depletion has little functional impact for mammary development. This may appear to be paradoxical, but it is consistent with recent data suggesting that the bipotential activity of mammary stem cells is not expressed during development; instead the growth of mammary ducts is mediated by division-competent cells of both lineages [11]. Indeed, mammary glands with mutations of integrin or Wnt signaling function are ostensibly normal, regardless of stem cell deficiency [11], [18], [21], [30].

Our results concur with the results of evaluation of another “non-essential” mammalian stem cell. Thus melanocytes are also extinguished (revealed as the graying of hair) after exposure to irradiation. This was attributed to the differentiation of melanocyte stem cells in their niche [4], and this result suggests that the niche could be the target of genotoxins. The niche for mammary stem cells has not been defined, but it is likely to include a source of Wnt ligands.

The cell cycle has also been shown to regulate stem cell responses, and for several other stem cell types during juvenile development, the response to genotoxin exposure is dominated by cell sacrifice. (In peri-pubertal ductal trees, we assume that mammary stem cells are in cycle). In contrast, in adult organs, after the same stem cell types have entered the G_zero_ phase of the cell cycle, the response changes to preserve function, at the expense of activating error-prone DNA repair mechanisms and increasing tumorigenic genetic changes [1]. For example, hair follicle bulge stem cells are damage resistant, show transient p53 activation, activate non-homologous end-joining, and are recruited to become precursor cells to basal cell carcinomas [5], [6].

A link between stem cells and tumor precursor cells is evident for some lineages but not for others.

For breast tissues, the only example of a cell-of-origin analysis for breast tumors has been extracted from the study of Brca1-deficient patients and mice. In this case, the cell of origin appears to be a luminal cell type [31], [32], and not a basal cell. Indeed, it is not known whether basal stem cells are ever recruited as tumor precursor cells. If they were, our results would predict that early exposure to genotoxins may be protective to subsequent oncogenic challenge.

This study examined the effect of peri-pubertal DMBA exposure, using an intraperitoneal administration of a single dose. We devised this as a challenge to dividing mammary stem cells, but it is not a commonly used protocol in comparison to the post-pubertal administration of DMBA to BALB/c mice by gavage. Whereas the protocol described here does not induce mammary tumors (for at least 9 months; data not shown), the post-pubertal gavage protocol is highly tumorigenic and selective for mammary tissues. Luminal cells show massive hyper-proliferation within weeks of the last dose of DMBA, they generate a robust ductal carcinoma *in situ*, and tumors develop in almost all BALB/c mice within 200 days (other strains are less susceptible [16], [20]). The gavage protocol is fractionated, and the fat-soluble DMBA is delivered via VLDL particles to concentrate in the post-pubertal mammary fat pad. Interestingly, we found that the gavage protocol did not reduce the stem cell activity by the same degree (approx. 20%; data not shown).

A previous report concluded that mammary progenitors are radiation-resistant [33]. Thus, after irradiation of mammary glands (2 Gy), the fraction of SP cells increased, and the mammosphere forming potential increased. Given the development of more specific markers of mammary luminal progenitors, it would be useful to revisit this conclusion (for example CD61, α2 integrin or c-kit can partly distinguish dividing and non-dividing luminal cells [34], [35], [36]). The data presented here relates to basal stem cells, and does not address the effect of DMBA exposure on luminal mammary progenitor cells. Clonogenicity of mammary epithelial cells in culture is highly species- and assay-dependent, and difficult to extrapolate between mouse, rat and human. However, a previous study of rat mammary epithelial cell populations suggested that clonogens were only radiosensitive in pre-pubertal development (a result that would correspond to the one reported here) [37], and also showed that the response of clonogenic cells was different for mutagens that did not induce double strand breaks [38].

Our results show that both basal and luminal cell types sense genotoxin exposure similarly (assayed by γH2AX focus assembly), and both activate proximal checkpoint proteins in common (p53 and Chk2). Diehn et al [23] noted that basal mammary epithelial cells in normal glands showed lower levels of reactive oxygen species (ROS) than luminal mammary epithelial cells, and found that cell death was highly contingent on culture media conditions (as we did, Fig. S3). This was also extended to basal and luminal cell equivalents in tumors, and data were presented to support the idea that higher endogenous levels of ROS (either basal or induced) conferred a higher susceptibility to death by ionizing radiation. However, our study suggests that the cell death outcome depends on the provision of relevant survival/mitogenic factors. Thus, in the presence of ectopic Wnt ligands, the sensitivity of basal cells to genotoxins was clear, and the physiology observed *in vitro* matched the outcome *in vivo*.

Another study of human tissues has shown that there are basal and luminal lineage-specific responses to DNA damage. After sorting human mammary epithelial cells into basal and luminal cells (using an antibody to CD10), Huper and Marks (2007) tested for cell lineage specific responses [22]. They also observed the activation of proximal checkpoint proteins (assaying γH2AX, p53 and Brca1), and found that resolution of Brca1-foci was quicker for basal cells, the transactivation targets for p53 were cell type specific (for example, 14-3-3σ was basal-specific) and basal cells re-entered the cell cycle after a transient arrest (whereas luminal cells durably arrested for more than 80 hours). Neither cell type showed significant levels of cell death.

We could not detect significant rates of cell death induced by this dose of DMBA exposure *in vivo*; instead the response to this agent includes a durable (though minor) hyperproliferation of MECs, focalized in “burst centers”. These centers may correlate with compensatory regenerative response centers, at sites of damage repair [39]. Growth stimuli activated by DNA damage repair mechanisms include hypomorphic function for Atm. Thus, surprisingly (given that Atm is essential to growth for most cell lineages), hypomorphic Atm function in mammary epithelial cell models generates a proliferative signal [40].

The phenotype of mammary glands after genotoxic exposure (reduced basal cell number and stem cell activity) was a remarkable phenocopy of glands with loss of function for Wnt signaling. This prompted our evaluation of Wnt signaling function in these glands. Interestingly, activation of Wnt signaling receptors (phospho-S^1490^ Lrp) was reduced in treated mammary glands, and this loss was durable for weeks, long after the proximal responses to genotoxic exposure were complete. Loss of Lrp activation clearly corresponded with the loss of mammary stem cell activity. In fact, measuring Lrp phosphorylation could act more generally as a proxy measure for the number of stem cells in a larger population, given that luminal cells do not typically express either Lrp receptor [17], [41], and Axin2-positive (Wnt signal reporter) are limited to rare basal cells in the adult ductal tree *in vivo* [42].

Note that Zeng and Nusse have shown that Wnt3a (200 ng/ml) supplementation maintains mammary stem cell activity *in vitro* [42]. Our study of mammary epithelial cell cultures shows that Wnt signaling supports the expansion of the basal epithelial lineage in general. When the basal cell subpopulation is expanded by adding Wnt ligands, these cells became highly sensitized to genotoxic exposure, implying that a specific chemosensitivity is induced by the basal cell response to Wnt signaling.

### Conclusions

We have shown that when Wnt signaling is active in cultured basal cells, including basal stem cells, they become highly sensitized to genotoxic exposure. A similar result has been also observed for melanocyte stem cell activity; this activity was also extinguished by genotoxic exposure (in fact stem cells differentiate *in situ*), and was also shown to be Wnt-dependent for maintenance and survival [43]. We believe therefore that this interaction may have general significance for the proper maintenance of stem cells in culture, especially as it relates to their use for regenerative medicine. We also propose that if this interdependence extends to include Wnt-dependent tumor cells (such as colorectal cancer cells), it would explain the efficacy of chemotherapeutics that induce double strand breaks. We further suggest that the minor phenotypes that accompany the elimination of adult somatic stem cell activity after genotoxic exposure could be largely invisible in mammals. However, an understanding of the history of environmental exposures for a given individual could explain differences in their subsequent regenerative activity, and also their susceptibility to tumor development.

## Supporting Information

Figure S1 **Gating trees for flow cytometric analysis of mammary epithelial cells.** (A) Mammary epithelial cells were prepared from vehicle- or DMBA-treated females (9–10 weeks of age) and fixed for analysis. Representative flow cytograms are shown, gated to eliminate debris (a,e; side scatter, SSC-A, versus forward scatter, FSC-A), to eliminate cell doublets and aggregates (b,f; DAPI area versus DAPI width gate), to eliminate non-epithelial cells (c,g; APC-CD45/CD31 versus forward scatter, FSC-A) and to analyze the remaining epithelial cells for their expression of CD24 and CD49f (d,h). (B) Fractions of cells going forward through each gate are shown.
(TIF)

Figure S2 **DMBA induces double strand DNA breaks, shown by the appearance of γH2AX, and DDR response activation. (A) Western blotting data; γH2AX activation.** As a cross-check of the specificity of immunolocalization results for γH2AX (Fig. 4A, S3), mammary epithelial cells (HC11, NMuMG or BALB/c MECs) were treated with various genotoxins, lysed and transferred to Western blots, probing for the appearance of γH2AX. The genotoxins administered were 10 Gy irradiation (1 hour), 2 µg/ml DMBA (24 hours; this is a pro-genotoxin requiring activation by metabolism, or vehicle, DMSO), *N*-ethyl-*N*-nitrosourea (1 hour after addition of 500 µg/ml ENU, a direct acting alkylating agent, or control PCB), or 20 µg/ml camptothecin (1 hour, camptothecin is a topoisomerase I inhibitor, or DMSO). Non-treated cells (NT) are also shown for comparison. (B) *pS^15^-p53 is specifically induced by DNA damage in mammary epithelial cells, whereas pS^20^-p53 is constitutively present*. HC11 mammary epithelial cells and primary mammary epithelial cells (MECs) were treated with ionizing radiation (IR, 10 Gy) and immuno-stained 1 hour later for the presence of *pS^15^-p53 and pS^20^-p53*. (C) *Constitutive nuclear staining of BRCA1.* BALB/c MECs were treated with DMBA or IR (as above), fixed and stained for BRCA1 (with a nuclear TOPRO counterstain). BRCA1 staining was ubiquitous and nuclear in all cells, irrespective of DNA damage. NT, non-treated.
(TIF)

Figure S3 **Evaluation of the DNA damage response (DDR) in basal and luminal epithelial cells after genotoxin administration.** (A) *Assembly of damage foci.* Mammary epithelial cells (MECs) were transferred to culture for 24 hours, and exposed to 2 µg/ml DMBA or vehicle control (DMSO). Samples were analyzed 24 or 48 hours thereafter. The assembly of γH2AX foci (green) were assayed for basal (K5-positive; blue; B) and luminal (K8-positive; red; L) epithelial cells (counterstained with TOPRO to visualize nuclei). A representative stain is shown, and lineage-specific DNA damage quantified below (n = 3, cell number scored = 1000 each). * *p*<0.05; Wilcoxon Rank Sum Test. (B, C) *DDR checkpoint activation.* Similar cultures were assayed for activation of p53 (B), using immunohistochemical staining of phospho-p53 (pS^15^-p53; green), or Chk2 (C), by immunohistochemical staining of phospho-Chk2 (pT68-Chk2; green). Representative stains of DDR activation are shown together with the quantitation of lineage-specific responses, as for (A). (D, E) *Lineage specific DDR responses.* Total MECs were transferred to culture, and treated with DMBA. 48 hours later, cells were fixed, counted and stained for their lineage markers (D). Cultures were depleted of almost 50% of luminal cells. We also considered the possibility that genotoxin exposure could modulate differentiation to change the cellular composition of mammary glands. To test this outcome, purified luminal and basal cell types were live sorted by flow cytometry, and placed separately into culture, with and without DMBA. 24 hours later, cells were fixed and stained. Mouse basal epithelial cells are bipotent in culture (Badders et al., 2009) but luminal cells are specified, and stay luminal in culture (E), as illustrated by these lineage marker stains. Quantitation of the number of cells of each type present confirmed that these cells were acutely sensitive *in vitro* to DMBA exposure (E). Basal cells in culture showed normal differentiation to luminal fates in the presence of DMBA, and these basal-derived luminal cells were also sensitive to cell death after DMBA exposure (quantitation of these cultures from n = 2; cell number scored = 500).
(TIF)
