# Supplementary figures and images for: Genotoxic Exposure during Juvenile Growth of Mammary Gland Depletes Stem Cell Activity and Inhibits Wnt Signaling
Source: PLoS One. 2012 Nov 21;7(11):e49902. doi: 10.1371/journal.pone.0049902 (PMC3503807; doi:10.1371/journal.pone.0049902)

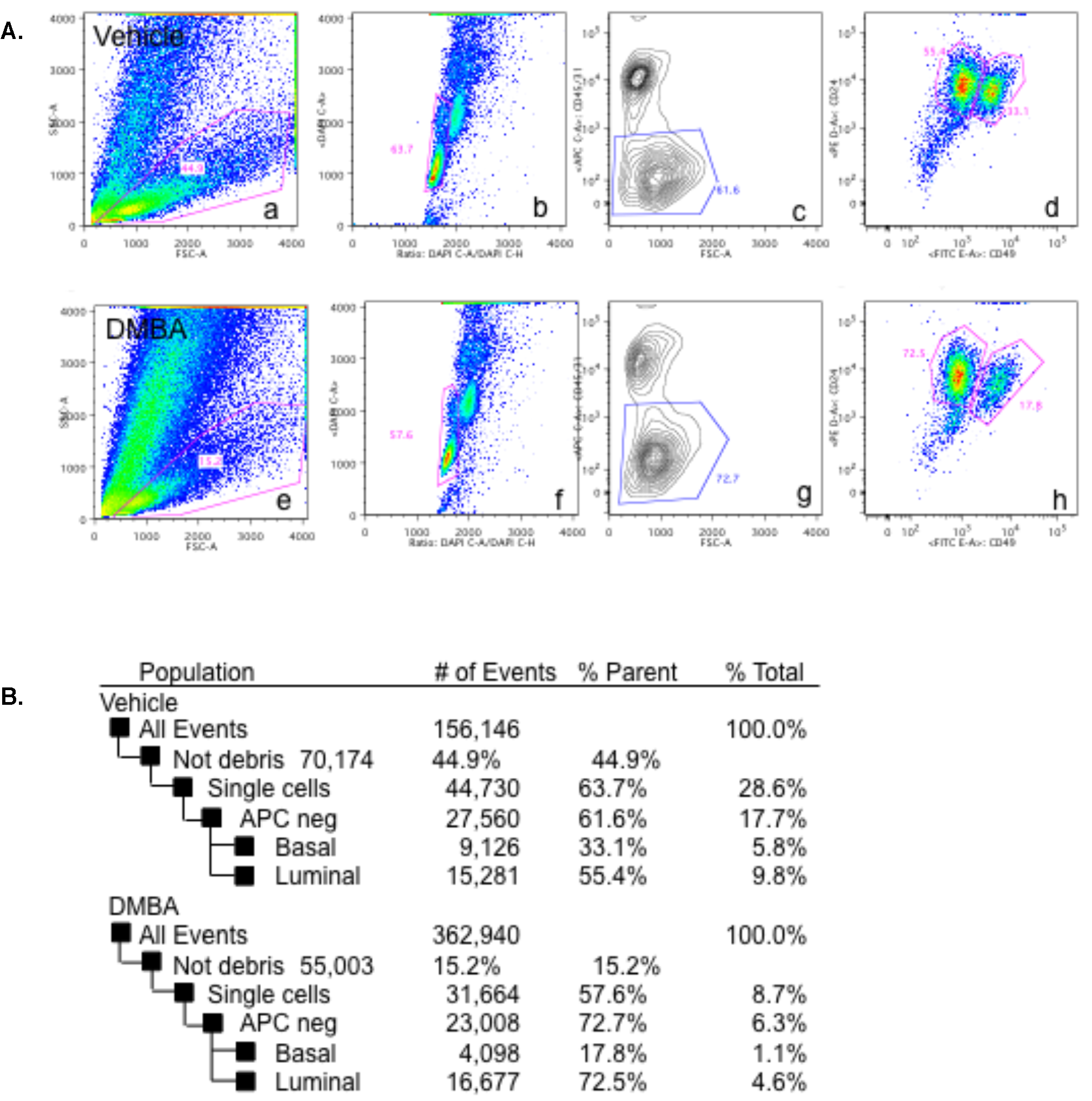

Supplement: Figure S1 — Gating trees for flow cytometric analysis of mammary epithelial cells. (A) Mammary epithelial cells were prepared from vehicle- or DMBA-treated females (9–10 weeks of age) and fixed for analysis. Representative flow cytograms are shown, gated to eliminate debris (a,e; side scatter, SSC-A, versus forward scatter, FSC-A), to eliminate cell doublets and aggregates (b,f; DAPI area versus DAPI width gate), to eliminate non-epithelial cells (c,g; APC-CD45/CD31 versus forward scatter, FSC-A) and to analyze the remaining epithelial cells for their expression of CD24 and CD49f (d,h). (B) Fractions of cells going forward through each gate are shown. (TIF) [file pone.0049902.s001.tif]

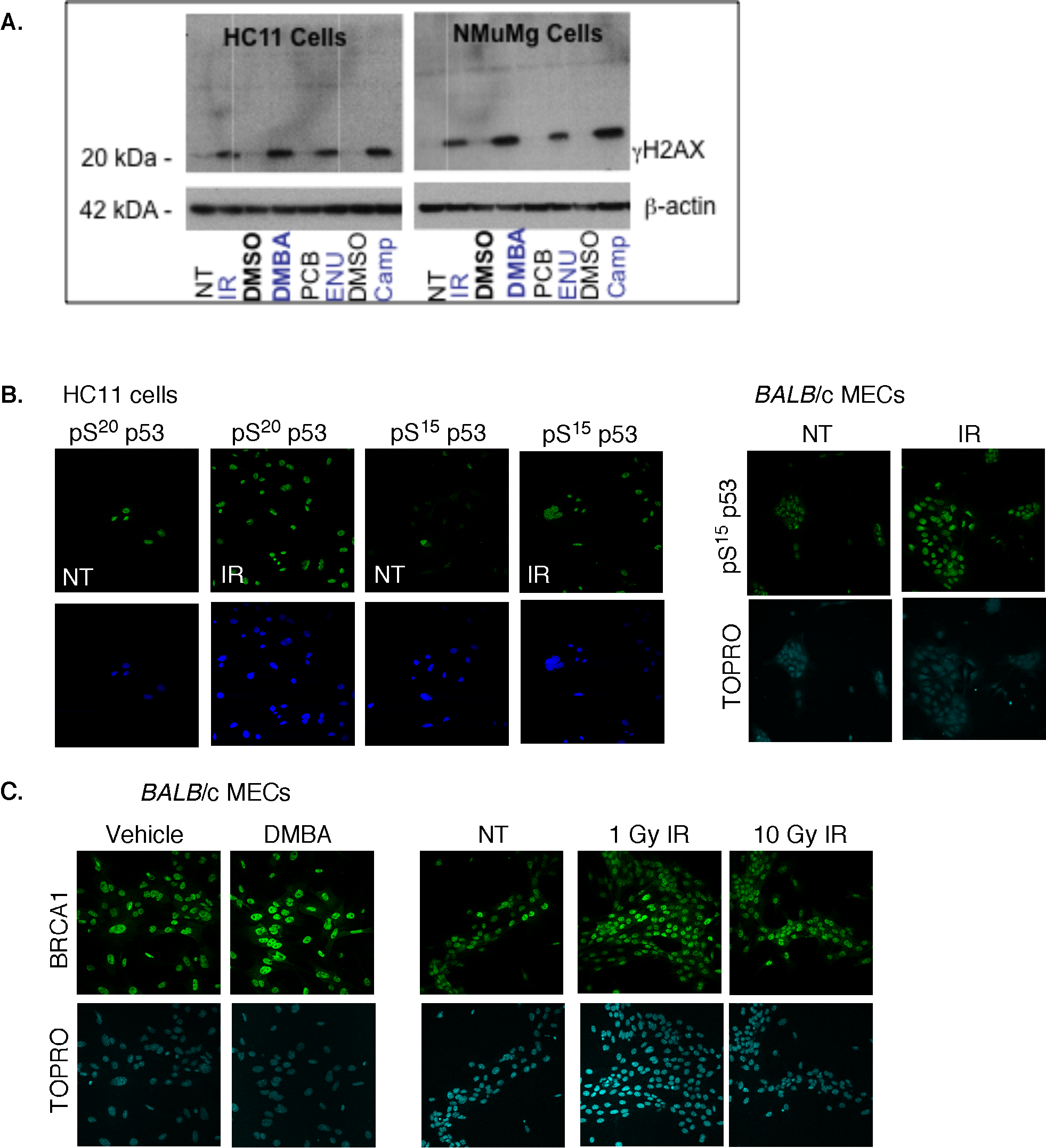

Supplement: Figure S2 — DMBA induces double strand DNA breaks, shown by the appearance of γH2AX, and DDR response activation. (A) Western blotting data; γH2AX activation. As a cross-check of the specificity of immunolocalization results for γH2AX (Fig. 4A, S3), mammary epithelial cells (HC11, NMuMG or BALB/c MECs) were treated with various genotoxins, lysed and transferred to Western blots, probing for the appearance of γH2AX. The genotoxins administered were 10 Gy irradiation (1 hour), 2 µg/ml DMBA (24 hours; this is a pro-genotoxin requiring activation by metabolism, or vehicle, DMSO), N-ethyl-N-nitrosourea (1 hour after addition of 500 µg/ml ENU, a direct acting alkylating agent, or control PCB), or 20 µg/ml camptothecin (1 hour, camptothecin is a topoisomerase I inhibitor, or DMSO). Non-treated cells (NT) are also shown for comparison. (B) pS15-p53 is specifically induced by DNA damage in mammary epithelial cells, whereas pS20-p53 is constitutively present. HC11 mammary epithelial cells and primary mammary epithelial cells (MECs) were treated with ionizing radiation (IR, 10 Gy) and immuno-stained 1 hour later for the presence of pS15-p53 and pS20-p53. (C) Constitutive nuclear staining of BRCA1. BALB/c MECs were treated with DMBA or IR (as above), fixed and stained for BRCA1 (with a nuclear TOPRO counterstain). BRCA1 staining was ubiquitous and nuclear in all cells, irrespective of DNA damage. NT, non-treated. (TIF) [file pone.0049902.s002.tif]

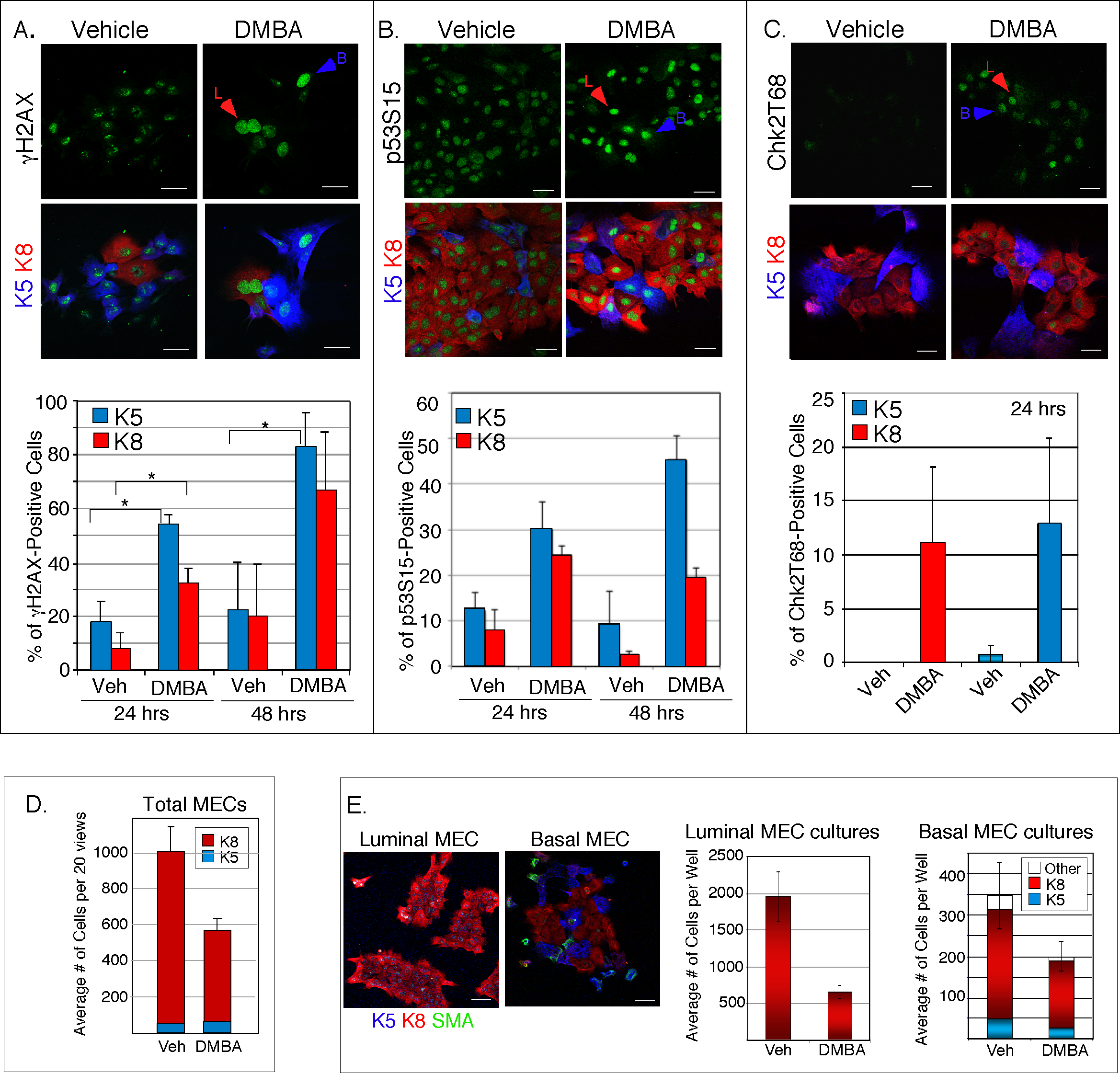

Supplement: Figure S3 — Evaluation of the DNA damage response (DDR) in basal and luminal epithelial cells after genotoxin administration. (A) Assembly of damage foci. Mammary epithelial cells (MECs) were transferred to culture for 24 hours, and exposed to 2 µg/ml DMBA or vehicle control (DMSO). Samples were analyzed 24 or 48 hours thereafter. The assembly of γH2AX foci (green) were assayed for basal (K5-positive; blue; B) and luminal (K8-positive; red; L) epithelial cells (counterstained with TOPRO to visualize nuclei). A representative stain is shown, and lineage-specific DNA damage quantified below (n = 3, cell number scored = 1000 each). * p<0.05; Wilcoxon Rank Sum Test. (B, C) DDR checkpoint activation. Similar cultures were assayed for activation of p53 (B), using immunohistochemical staining of phospho-p53 (pS15-p53; green), or Chk2 (C), by immunohistochemical staining of phospho-Chk2 (pT68-Chk2; green). Representative stains of DDR activation are shown together with the quantitation of lineage-specific responses, as for (A). (D, E) Lineage specific DDR responses. Total MECs were transferred to culture, and treated with DMBA. 48 hours later, cells were fixed, counted and stained for their lineage markers (D). Cultures were depleted of almost 50% of luminal cells. We also considered the possibility that genotoxin exposure could modulate differentiation to change the cellular composition of mammary glands. To test this outcome, purified luminal and basal cell types were live sorted by flow cytometry, and placed separately into culture, with and without DMBA. 24 hours later, cells were fixed and stained. Mouse basal epithelial cells are bipotent in culture (Badders et al., 2009) but luminal cells are specified, and stay luminal in culture (E), as illustrated by these lineage marker stains. Quantitation of the number of cells of each type present confirmed that these cells were acutely sensitive in vitro to DMBA exposure (E). Basal cells in culture showed normal differentiation to lumina [file pone.0049902.s003.tif]
